# Menopausal hormone therapy after ovarian cancer: A 10‐year survival analysis in premenopausal women

**DOI:** 10.1111/aogs.70242

**Published:** 2026-05-20

**Authors:** Åsa Ehlin von Kartaschew, Pernilla Dahm‐Kähler, Kenny Rodriguez Wallberg, Erik Holmberg, Angelique Flöter‐Rådestad

**Affiliations:** ^1^ Livio Fertility Center Stockholm Sweden; ^2^ Department of Women's and Children's Health, Division of Neonatology, Obstetrics, Gynecology and Reproductive Health Karolinska Institute Stockholm Sweden; ^3^ Institute of Clinical Sciences, Sahlgrenska Academy at University of Gothenburg Gothenburg Sweden; ^4^ Department of Reproductive Medicine, Division of Gynecology and Reproduction Karolinska University Hospital Stockholm Sweden; ^5^ Department of Oncology‐Pathology, Laboratory of Translational Fertility Preservation Karolinska Institute Stockholm Sweden; ^6^ Department of Oncology, Institute of Clinical Sciences, Sahlgrenska Academy University of Gothenburg Gothenburg Sweden; ^7^ Regional Cancer Centre Western Sweden Sahlgrenska University Hospital Gothenburg Sweden; ^8^ Department of Hereditary Cancer Karolinska University Hospital Stockholm Sweden

**Keywords:** borderline ovarian tumor, epithelial ovarian cancer, menopausal hormone therapy, non‐epithelial ovarian cancer, ovarian cancer, overall survival, surgical menopause

## Abstract

**Introduction:**

Premenopausal women treated with bilateral salpingo‐oophorectomy (BSO) for ovarian cancer (OC) enter surgical menopause. Our objective was to evaluate whether postoperative systemic menopausal hormone therapy (MHT) after BSO due to treatment for epithelial ovarian cancer (EOC), non‐epithelial ovarian cancer (NEOC), or borderline ovarian tumor (BOT) has an impact on long‐term overall survival (OS).

**Material and Methods:**

A nationwide Swedish population‐based cohort study of women aged 18–50 diagnosed with OC (FIGO stage I–IV) and treated with BSO between 2008 and 2015, identified from The Swedish Quality Register for Gynecological Cancer. Exposure to postoperative MHT from 1 month before to 5 years after surgery was assessed by linking the cohort to the National Prescribed Drug Register to capture drug dispensing data. Standardized OS was estimated for MHT users and non‐users, adjusted for covariates included in multivariable models. EOC, NEOC, and BOT were analyzed separately.

**Results:**

A cohort of 779 premenopausal women with a median age of 45 years at BSO, of whom 472 had an EOC, 68 a NEOC and 239 a BOT, was identified. Median follow‐up in the total cohort was 11.7 (9.6–13.6) years. Among women with EOC, 33% were postoperative MHT users. For the EOCs, the 10‐year OS for postoperative MHT users versus non‐users was 52.4% (95% CI 46.7–58.8) and 51.7% (95% CI 47.4–56.4), respectively. No significant difference in 10‐year OS was found between postoperative MHT users and non‐users (HR 0.97; 95% CI 0.73–1.30; *p* = 0.84). Among 367 women with EOC and no residual disease at surgery, there was no difference in 10‐year OS between postoperative MHT users and non‐users (HR 1.00; 95% CI 0.70–1.43; *p* = 0.98). For women with a BOT, the 10‐year OS was 96.2% (95% CI 91.6–98.2) for postoperative MHT users versus 95.3% (95% CI 87.8–98.2) for non‐users. Among women with a NEOC, OS was not analyzed due to the few events.

**Conclusions:**

In this nationwide cohort, postoperative MHT use after surgically treated OC was not associated with impaired 10‐year survival. These findings offer reassurance about the safety of MHT in young ovarian cancer survivors.

## Graphical abstract

Among premenopausal women surgically treated for ovarian cancer, no difference in 10‐year overall survival was observed between postoperative MHT users and non‐users. Young ovarian cancer survivors should be recommended MHT after surgically induced menopause.

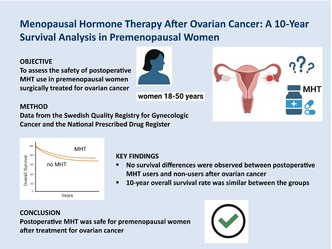

**Abbreviations**
BOT: borderline ovarian tumor
BSO: bilateral salpingo‐oophorectomy
EOC: epithelial ovarian cancer
MHT: menopausal hormone therapy
NEOC: non‐epithelial ovarian cancer
OC: ovarian cancer
OS: overall survival

**Key message**

Among premenopausal women surgically treated for ovarian cancer, postoperative MHT use was safe. No difference in 10‐year overall survival rates was seen between MHT users and non‐users. Young ovarian cancer survivors should be recommended MHT following surgically induced menopause.

## 1 INTRODUCTION

Advances in the treatment of ovarian cancer (OC) have increased survival rates.^1^ Approximately 20% of all women are premenopausal at the time of OC diagnosis.^2^ A majority will undergo treatment, most often surgery, including bilateral salpingo‐oophorectomy (BSO) in combination with chemotherapy, resulting in premature menopause. Surgical menopause leads to an abrupt onset of menopausal symptoms with a negative effect on quality of life.^3^ There are also well‐established negative long‐term health consequences of premature menopause, such as osteoporosis, cardiovascular disease, and increased overall mortality.^4^, ^5^, ^6^, ^7^ Menopausal hormone therapy (MHT) effectively relieves these symptoms and improves long‐term health. An increasing number of cancer survivors and concerns about quality of life and recurrences have led to a need to address the safety of postoperative MHT use in young ovarian cancer survivors.^8^

Use of MHT before diagnosis has been associated with a small increased risk of developing epithelial ovarian cancer (EOC).^9^ Thus, there is a concern that MHT use after OC would increase the risk of recurrence. Studies have evaluated the safety of MHT after EOC, suggesting that postoperative MHT does not negatively impact overall survival (OS) or recurrence risk; on the contrary, MHT improved OS.^10^, ^11^, ^12^, ^13^, ^14^, ^15^, ^16^, ^17^ However, arguments have been raised about a possible selection bias toward women with better prognosis among MHT users in these studies, and the data are too limited to support or refute MHT use in this population.^8^ A gap in the literature concerns premenopausal women. Despite the potential benefits of MHT for quality of life and long‐term health, few studies have evaluated its safety in premenopausal ovarian cancer survivors, and OS data remain scarce. Furthermore, cohort studies analyzing OS in women receiving MHT or not after OC treatment stratified by histopathological tumor type, stage, and duration of MHT treatment, as well as studies with longer follow‐up, are needed. For borderline ovarian tumors (BOT), one study found no association between postoperative MHT and OS, and, to our knowledge, no data exist for non‐epithelial ovarian cancer (NEOC).^10^ Notably, the safety of postoperative MHT use in premenopausal ovarian cancer survivors remains largely unstudied across all histological subtypes.

Menopausal symptoms among OC survivors are common, yet reported rates of MHT use after treatment remain low.^18^, ^19^ In a previous study, we investigated the dispensing rate of MHT following surgical menopause after OC treatment. We found that the MHT dispensing rate was only 62% in women < 40 years within the first year after BSO.^20^ The primary aim of this study was to evaluate whether postoperative MHT use after surgery for EOC, NEOC, or BOT in premenopausal women affects OS.

## 2 MATERIAL AND METHODS

### 2.1 Study design and population

In this nationwide population‐based cohort study, all women diagnosed with OC at ages 18–50 years from January 1, 2008, to December 31, 2015 were retrieved from the Swedish Quality Register for Gynecological Cancer (SQRGC). All women who had a BSO performed as part of treatment for EOC, NEOC, or BOT, hereafter referred to as OC, were identified. Exclusion criteria were former or current breast‐ or endometrial cancer, due to contraindication for MHT use. Within this defined cohort, the postoperative MHT use and its impact on OS were analyzed separately for each histological subtype. The registers and methods used for analyses of the cohort have previously been described in detail.^20^

The SQRGC, established in 2008, prospectively and consequently collects data on clinical, surgical, oncological variables, pathology reviews, as well as mortality data.^21^, ^22^

Information on tumor location and classification was obtained from the SQRGC and defined according to the World Health Organization criteria.^23^ Serous EOC was not divided into high‐grade or low‐grade since this classification was introduced during the study period and missing during 2008–2010. Fallopian tube cancer and EOC were grouped and referred to as EOC. The BOTs were classified as mucinous or serous since more specific details were missing in 2008. Tumor stage was defined according to the FIGO classification system.^24^

### 2.2 Data sources

All citizens in Sweden are assigned a 12‐digit personal identification number. This number was used to obtain further data on the individuals in the study cohort from the Swedish National Cancer Register (NCR), the National Cause of Death Register, the National Patient Register (NPR), and the National Prescribed Drug Register.^25^ All four registers are maintained by the National Board of Health and Welfare.

The NPR contains information on all completed inpatient stays, nationwide since 1987, as well as data on patients treated by doctors in specialized outpatient care since 2001. Chemotherapy was defined as adjuvant chemotherapy registered in the SQRGC and/or the International Classification of Diseases (ICD)‐10 codes for Z51.1 (chemotherapeutic treatment for tumor) and/ or KVÅ DT116 (intravenous chemotherapy) extracted from the NPR. The NPR codes were added to avoid misclassification bias. The National Cause of Death register and the Swedish Population Register, maintained by the Swedish Tax Agency, were used to ensure follow‐up, emigration, and date of death.

The prognostic factors, including age, FIGO stage, histological subtype, and complete cytoreduction to no residual disease, were included in the analyses. Women were subgrouped by age and categorized as ≤ 44 or ≥ 45, due to MHT being most important for women with premature and early menopause. Additional analysis was conducted for the EOC subcohort, where complete cytoreduction to no residual disease was achieved at surgery, referred to as “no residual disease.”

### 2.3 Assessment of menopausal hormone therapy

To assess MHT exposure, data on postoperative MHT dispensing were obtained from the National Prescribed Drug Register and used as a proxy for MHT use.^26^ We included dispensing 30 days before BSO to cover prescribed MHT before surgery. This register is fully automated and records all dispensed prescriptions from pharmacies in Sweden. MHT can only be obtained through a prescription from a physician. Codes from the Anatomical Therapeutic Classification system (ATC‐codes) were used to define hormone therapy‐codes. Data for systemic hormone therapy, defining MHT dispensing, was obtained for contraceptives containing estrogen (G03A), oral and transdermal estrogen (G03C), and systemic progesterone and estrogen in combination (G03F). Products for vaginal estrogen treatment were excluded from the analyses.

### 2.4 Statistical analysis

In the descriptive analysis, women were categorized as *MHT users within 5 years* if they had at least one dispensing of MHT from the date of surgery up to 5 years postoperatively. Women without any such dispensing were classified as *MHT non‐users within 5 years*. Comparisons between MHT exposure groups were performed using Fisher's exact test for categorical variables and the Wilcoxon rank‐sum test for continuous variables. Variables with undefined, missing, or not applicable categories were excluded from statistical comparisons.

In time‐to‐event analyses of cumulative incidence and OS, women were initially classified as *MHT non‐users* and reclassified as *MHT users* from the date of first MHT dispensing onwards. Consequently, women could contribute person‐time to both exposure groups. Cumulative incidence functions accounting for the competing risk of death were estimated using the stcompet command in Stata. Time was measured from the date of surgery to the first occurrence of MHT dispensing (event), death (competing event), emigration (censoring), or last follow‐up in February 2024 (censoring). Five‐year cumulative incidence curves were constructed to visualize the cumulative proportion of MHT use and mortality over time.

Uni‐ and multivariable associations with OS were analyzed using flexible parametric survival models.^27^ Cause‐specific hazard ratio (HR), 95% confidence interval (CI), and two‐sided *p*‐values were reported. We evaluated the proportional hazards assumption by analyzing the Schoenfeld residuals.^28^, ^29^ In cases where the assumption was violated, the reported hazard ratios should be considered as average effects over the follow‐up duration rather than instantaneous relative risks. Standardized survival curves were estimated for MHT users and non‐users, adjusted for covariates included in the multivariable models, to reflect average survival in the study population. The regression models used for these survival curves used splines for time‐varying covariates when the proportional hazards assumption was violated. The 5‐ and 10‐year OS with 95% confidence interval were estimated from the standardized curves. Standardization was performed using the standsurv command in Stata.^30^

All statistical analyses were performed using Stata (StataCorp. 2025. *Stata: Release 18.5. Statistical Software*. College Station, TX: StataCorp LLC). A two‐sided *p*‐value ≤ 0.05 was considered statistically significant.

## 3 RESULTS

**FIGURE 1:**
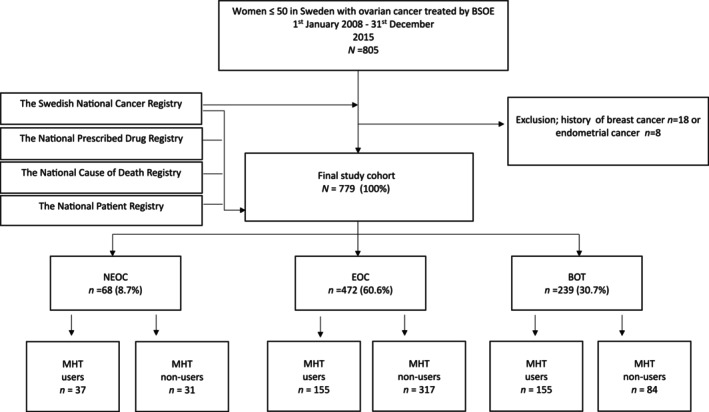
Flowchart of study population of women with ovarian cancer ≤50 years treated by bilateral salpingo‐oophorectomy. BOT, borderline ovarian tumor; BSO, bilateral salpingo‐oophorectomy; EOC, epithelial ovarian cancer; MHT, menopausal hormone therapy; NEOC, non‐epithelial ovarian cancer.

We identified 805 women between 18 and 50 years of age who had a BSO performed due to OC during the study period. After exclusion of 26 women with a prior history of breast or endometrial cancer, the cohort consisted of 779 women who met the inclusion criteria (Table 1, Figure 1).

**TABLE 1:** Patient, tumor, and treatment characteristics of ovarian cancer in the total cohort stratified by MHT use within 5 years.

	Total cohort	MHT‐user within 5 years total cohort	
		No	Yes
	*N* = 779	*n* = 432 (55.5%)	*n* = 347 (44.5%)
Age at surgery (years), median (IQR)	45.0 (39.1–48.0)	46.9 (43.0–49.0)	42.0 (36.7–46.0)
Age at surgery (years), age group			
18–29	52	20 (38.5)	32 (61.5)
30–34	43	13 (30.2)	30 (69.8)
35–39	104	29 (27.9)	75 (72.1)
40–44	181	88 (48.6)	93 (51.4)
45–50	399	282 (70.7)	117 (29.3)
Year of surgery			
2008	81	42	39
2009	98	58	40
2010	118	66	52
2011	92	51	41
2012	65	33	32
2013	102	57	45
2014	117	67	50
2015	106	58	48
FIGO stage			
I	443	218 (49.2)	225 (50.8)
II	65	41 (63.1)	24 (36.9)
III	214	133 (62.1)	81 (37.9)
IV	57	40 (70.2)	17 (29.8)
Subtype histology			
EOC serous	262	178 (67.9)	84 (32.1)
EOC mucinous	61	37 (60.7)	24 (39.3)
EOC endometroid	84	61 (72.6)	23 (27.4)
EOC clear cell	50	29 (58.0)	21 (42.0)
EOC other	15	12 (80.0)	3 (20.0)
NEOC germ cell	22	4 (18.2)	18 (81.8)
NEOC sex cord‐stromal cell	46	27 (58.7)	19 (41.3)
BOT serous	61	18 (29.5)	43 (70.5)
BOT mucinous	67	25 (37.3)	42 (62.7)
BOT other	111	41 (36.9)	70 (63.1)
Type of surgery			
Primary debulking surgery (PDS)	605	356	249
Interval debulking surgery (IDS)	9	4	5
Re‐staging	142	61	81
Undefined/missing	23	11	12
Complete cytoreduction to no residual disease			
Yes	638	341 (53.4)	297 (46.6)
No	90	64 (71.1)	26 (28.9)
Undefined/missing/not applicable	51	27 (52.9)	24 (47.1)
Hysterectomy			
No	87	41	46
Yes	690	389	301
Undefined	2	2	0
Adjuvant chemotherapy			
Yes	461	282 (61.2)	179 (38.8)
No	318	150 (47.2)	168 (52.8)
Time to first MHT use (months), median (IQR)	1.7 (0.2–6.4)		1.7 (0.2–6.4)
Follow‐up censored, median (IQR), years	11.7 (9.6–13.6)	11.7 (9.6–13.5)	11.7 (9.6–13.6)
Follow‐up death, median (IQR), years	3.5 (2.1–5.5)	3.2 (1.9–5.4)	4.6 (2.7–6.1)
Number of deaths/censored within 5 years			
Death	160	119 (74.4)	41 (25.6)
Censored	619	313 (50.6)	306 (49.4)
Menopausal hormone therapy			
Total number of dispensations, *n*			4635
Median number of dispensations per woman, median (IQR)			15 (5–20)
Number of dispensations per person years			3.0
Type of MHT dispensed, *n* (%)			
Estrogen only			4273 (92.2)
Tibolone			76 (1.6)
Estrogen and progesterone in combination			286 (6.2)
Route of administration, *n* (%)			
Oral			2686 (58)
Transdermal			1949 (42)

*Note*: Total cohort; includes epithelial ovarian cancer, non‐epithelial ovarian cancer, and borderline ovarian tumor. Data are presented as median (IQR) for continuous measures, and No. (row%) for categorical measures, except MHT dispensed and route of administration (column %).

Abbreviations: BOT, borderline ovarian tumor; EOC, epithelial ovarian cancer; MHT, menopausal hormone therapy; NEOC, non‐epithelial ovarian cancer.

In the total cohort, 44.5% (*n* = 347) were MHT users and 55.5% (*n* = 432) non‐users within 5 years after surgery (Table 1). Another 12 women became users after 5 years. Median time to initiation of MHT use after surgery was 1.7 (0.2–6.4) months. The number of dispensations per woman‐years was 3.0, and the median number (IQR) of dispensations was 15 (5–20). The type of MHTs dispensed within the first 5 years after surgery was: 92.2% estrogen only, 1.6% Tibolone, and 6.2% estrogen and progesterone in combination. Of them, 58% were oral and 42% were transdermal preparations.

### 3.1 Postoperative MHT use in women with EOC, NEOC and BOT

**FIGURE 2:**
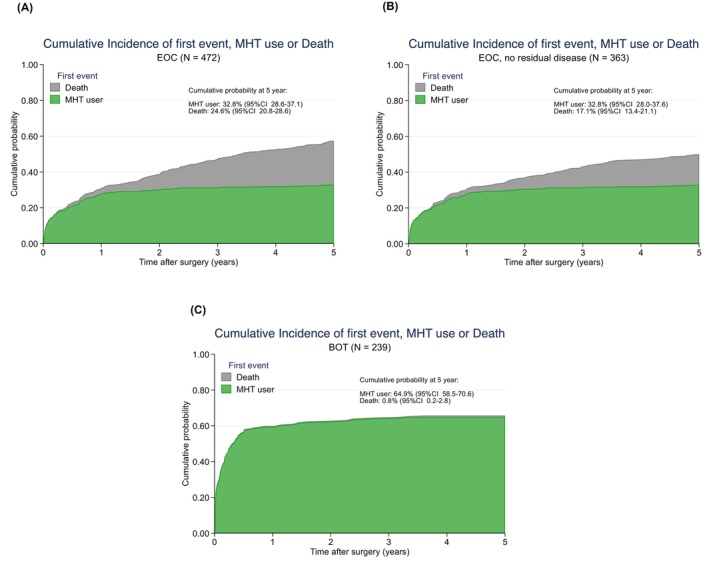
The cumulative incidence rate for first event, MHT use or death, in the study population; (A) Epithelial ovarian cancer. (B) Epithelial ovarian cancer with no residual disease at surgery. (C) Borderline ovarian tumor. BOT, borderline ovarian tumor; CI, confidence interval; EOC, epithelial ovarian cancer; MHT, menopausal hormone therapy; NEOC, non‐epithelial ovarian cancer.

A majority of women, 60.6% (*n* = 472), were diagnosed with an EOC, 8.7% (*n* = 68) with a NEOC, and 30.7% (*n* = 239) with a BOT. Of the women with an EOC, 32.8% (*n* = 155) were MHT users within 5 years after surgery (Table 2). MHT users were significantly younger than the non‐users (*p* < 0.001). No other differences in baseline data were seen between the MHT users and non‐users with an EOC. Of the women with an EOC, 77.8% (*n* = 367) had no residual disease at surgery (Table S1). Here, 34.1% (*n* = 125) were MHT users within 5 years after surgery. As in the total EOC cohort, age was the only significant difference in baseline data between the MHT users and the non‐users with no residual disease (*p* < 0.001). Among women with a NEOC and a BOT, the MHT use within 5 years was 54.4% (*n* = 37) and 64.9% (*n* = 155), respectively (Table S2). The cumulative incidence of the first event, MHT use or death among the women diagnosed with an EOC, EOC with no residual disease or a BOT, are shown in Figure 2A–C.

**TABLE 2:** Patient, tumor, and treatment characteristics of epithelial ovarian cancer, stratified by MHT use within 5 years.

	Total EOC	MHT‐user within 5 years EOC		*p* ^a^
		No	Yes	
	*n* = 472	*n* = 317 (67.2%)	*n* = 155 (32.8%)	
Age at surgery	45.0 (41.0–48.2)	47.0 (43.0–49.0)	42.0 (37.3–46.0)	< 0.001
Age at surgery				< 0.001
18–29	20	12 (60.0)	8 (40.0)	
30–34	21	7 (33.3)	14 (66.7)	
35–39	58	24 (41.4)	34 (58.6)	
40–44	110	66 (60.0)	44 (40.0)	
45–50	263	208 (79.1)	55 (20.9)	
Year of surgery				0.91
2008	52	33	19	
2009	59	40	19	
2010	69	49	20	
2011	51	36	15	
2012	31	23	8	
2013	69	46	23	
2014	73	45	28	
2015	68	45	23	
FIGO stage				0.10
I	180	109 (60.6)	71 (39.4)	
II	52	39 (75.0)	13 (25.0)	
III	185	129 (69.7)	56 (30.3)	
IV	55	40 (72.7)	15 (27.3)	
Subtype histology				0.26
EOC Serous	262	178 (67.9)	84 (32.1)	
EOC Mucinous	61	37 (60.7)	24 (39.3)	
EOC Endometroid	84	61 (72.6)	23 (27.4)	
EOC Clear Cell	50	29 (58.0)	21 (42.0)	
EOC Other	15	12 (80.0)	3 (20.0)	
Type of surgery				0.057
Primary debulking surgery (PDS)	385	268	117	
Interval debulking surgery (IDS)	8	4	4	
Re‐staging	67	38	29	
Undefined/missing	12	7	5	
Complete cytoreduction to no residual disease				0.20
Yes	367	242 (65.9)	125 (34.1)	
No	87	64 (73.6)	23 (26.4)	
Undefined/missing/not applicable	18	11 (61.1)	7 (38.9)	
Hysterectomy				0.070
No	38	20	18	
Yes	432	295	137	
Undefined	2	2	0	
Adjuvant chemotherapy				0.79
Yes	396	267	129	
No	76	50	26	
Time to first MHT use (months), median (IQR)	2.5 (0.4–8.4)		2.5 (0.4–8.4)	
Follow‐up censored, median (IQR), years	11.1 (9.4–13.6)	11.1 (9.5–13.5)	11.1 (9.3–13.6)	0.96
Follow‐up death, median (IQR), years	3.4 (2.1–5.4)	3.2 (1.9–5.3)	4.5 (2.6–5.6)	0.039
Number of deaths/censored within 5 years				0.022
Death	156	116 (74.4)	40 (25.6)	
Censored	316	201 (63.6)	115 (36.4)	

*Note*: Data are presented as median (IQR) for continuous measures, and No. (row %) for categorical measures, except MHT dispensed and route of administration (column %).

Abbreviations: EOC, epithelial ovarian cancer; MHT, menopausal hormone therapy.

^a^ Comparisons between MHT groups were performed using Fisher's exact test for categorical variables and the Wilcoxon rank‐sum test for continuous variables. Undefined, missing, or not applicable categories were excluded from statistical comparisons.

### 3.2 Survival in EOC following postoperative MHT use

**FIGURE 3:**
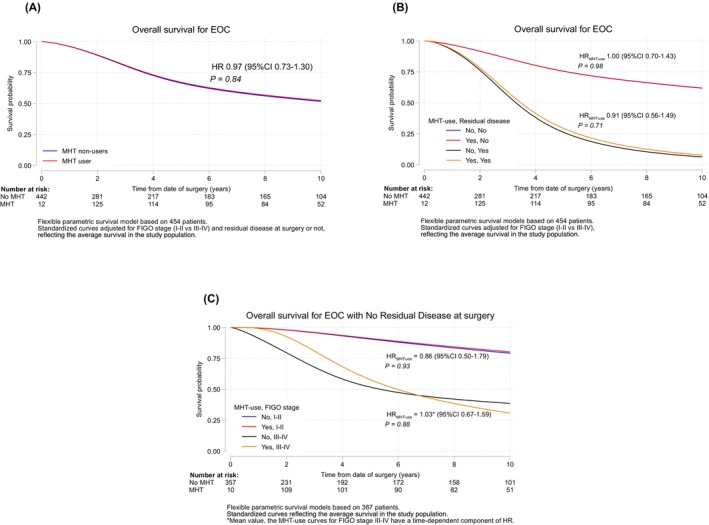
The 10‐year overall survival rate in women 18–50 years for; (A) Epithelial ovarian cancer (EOC), MHT use versus MHT non‐use, (B) epithelial ovarian cancer (EOC) divided by no residual disease *or* residual disease at surgery, MHT use versus non MHT use, (C) epithelial ovarian cancer with no residual disease at surgery, MHT use versus non‐MHT use. CI, confidence interval; EOC, epithelial ovarian cancer; FIGO, International Federation of Gynecology and Obstetrics; MHT, menopausal hormone therapy.

Survival was analyzed among the 472 women diagnosed with an EOC. The number of deaths within the first 5 years was 156. In the multivariable regression analysis, only FIGO stage and complete cytoreduction to no residual disease at surgery were significant predictors of OS, whereas age, subtype histology, and MHT use were not (Table 3). In the predictions from the regression model, adjusted for FIGO stage I‐II versus III‐IV and residual disease or not at surgery, for women with an EOC (*n* = 454), the 5‐year OS for the postoperative MHT users was 67.1% (95% CI 61.9–72.8) versus 66.5% (95% CI 62.7–70.6) for the non‐users. The 10‐year OS for the postoperative MHT users versus the non‐users was 52.4% (95% CI 46.7–58.8) and 51.7% (95% CI 47.4–56.4), respectively. Among women with an EOC, there was no significant difference in 10‐year OS between the postoperative MHT users and the non‐users; HR 0.97, 95% CI 0.73–1.30; *p* = 0.84 (Figure 3A).

**TABLE 3:** Flexible parametric survival regression analysis of epithelial ovarian cancer with 10‐year overall survival as the endpoint (*n* = 454).

	Univariable HR (95% CI)	*p*	Multivariable HR (95% CI)	*p*
MHT user				
No	1.0		1.0	
Yes	0.95 (0.71–1.26)	0.717	0.99 (0.74–1.32)	0.930
Age				
≤ 44	1.0			
45–50	1.01 (0.77–1.32)	0.932		
FIGO stage				
I–II	1.0^a^		1.0	
III–VI	6.25 (4.50–8.68)	< 0.001	4.78 (3.37–6.78)	< 0.001
Histology				
Serous	1.0			
Non‐serous	0.37 (0.27–0.50)	< 0.001		
Residual disease				
No	1.0^a^		1.0	
Yes	4.83 (3.63–6.41)	< 0.001	2.50 (1.85–3.37)	< 0.001

Abbreviations: CI, confidence interval; MHT, menopausal hormone therapy.

^a^ The proportional hazards assumption was violated.

### 3.3 Survival in BOT and NEOC following postoperative MHT use

Among the women with a BOT, a total of 3 deaths were registered at the 5‐year follow‐up and 10 deaths at the 10‐year follow‐up. The 10‐year OS was 96.2% (95% CI 91.6–98.2) for the postoperative MHT users versus 95.3% (95% CI 87.8–98.2) for the non‐users. Of the 68 women diagnosed with an NEOC, only one death occurred among the postoperative MHT users at the 10‐year follow‐up. Therefore, OS was not analyzed in this group.

### 3.4 Subgroup analysis of survival in EOC following postoperative MHT use

In the subgroup survival analysis among women with EOC stratified by the presence or absence of residual disease, no significant difference in 10‐year OS was found between postoperative MHT users and non‐users among those with no residual disease; HR 1.00 (95% CI 0.70–1.43); *p* = 0.98 (Figure 3B). Nor was there any difference in OS among those with residual disease and postoperative MHT use or not; HR 0.91 (95% CI 0.56–1.49); *p* = 0.71 (Figure 3B). Furthermore, in the subanalysis of the 367 women with no residual disease at surgery, no difference in 10‐year OS between the postoperative MHT users and non‐users was found among the women with a FIGO stage I or II; HR 0.86 (95% CI 0.50–1.79); *p* = 0.93. Nor did we find any difference in OS between women diagnosed with a FIGO III or IV and no residual disease, in relation to postoperative use of MHT; HR 1.03 (95% CI 0.67–1.59); *p* = 0.88 (Figure 3C).

## 4 DISCUSSION

In this nationwide population‐based study, including 779 women aged 50 years or younger treated with BSO for OC, and with a median follow‐up of 11.7 years, we found that postoperative use of MHT was not associated with impaired survival. Among women with EOC, no significant differences in OS were observed between MHT users and non‐users. Moreover, in the subgroup of women with EOC and no residual disease at the time of surgery, survival outcomes were similar regardless of MHT use. For women diagnosed with BOT, our findings did not indicate that MHT use after surgery was harmful. To our knowledge, this is the largest cohort study to date assessing the safety of MHT use in premenopausal women following surgery for OC.

Baseline was set to date for BSO, the time when women become postmenopausal and MHT use is indicated. Current national, as well as international, guidelines conclude that MHT can be offered to premenopausal women after OC treatment except for women with low‐grade serous carcinomas, endometroid, or granulosa cell tumors, since they are likely to be hormone‐sensitive.^31^, ^32^ However, in our prior publication, we found a low dispensing rate among young OC survivors, indicating a low rate of use.^20^ One may speculate that there may be a fear of increasing the risk of recurrence of disease, as well as concerns for thrombosis and/or inducing new hormone‐dependent cancers, influencing both the profession and the women, and thus both the prescription as well as dispensing of MHT following OC.

Among women with EOC, there was no difference in survival between MHT and non‐users. Baseline data between the MHT groups differed only in age, indicating that younger women were more likely to be prescribed and dispensed MHT. However, younger age was not associated with increased survival in the multivariable regression analysis, whereas stage and no residual disease were. The higher proportion of EOC cases classified as FIGO stages I and II is probably due to the cohort being limited to women surgically treated for OC and exclusively at the ages ≤ 50 years. The 5‐year OS in the EOC cohort was approximately 67% for both MHT users and non‐users. Thus, the higher proportion of early‐stage disease likely also explains the favorable survival rates found in our EOC cohort.^8^

Our results are congruent with other studies on postoperative MHT use and EOC survival, although in cohorts with various ages. Two RCTs, including 125 and 75 women, respectively, showed no negative effect of MHT use and survival after EOC treatment.^11^, ^12^ Three retrospective cohort studies did not show a decrease in overall survival after MHT use in EOC.^17^, ^33^, ^34^ In addition, a prospective Australian study by Na et al., MHT use after treatment was not associated with a difference in OS.^19^ However, a third RCT from Eeles et al. including 150 women, showed improved OS after MHT use.^16^ Further, a prospective Swedish study including 649 women with EOC aged 50–74 also showed that OS increased among MHT users.^10^ Moreover, in one Korean study by Ji et al. including 1784 women 60 years or younger MHT use was associated with increased OS survival.^15^ However, data on FIGO stage, subtype histology and residual disease were lacking in this study.

Physicians may be more prone to prescribe MHT to women with an overall good health status and good prognosis. Therefore, we further examined the effect on MHT use and non‐use in women with no residual disease at surgery. However, we found no difference between users and non‐users among women diagnosed with an EOC and no residual disease in the model also adjusting for FIGO stage.

Non‐serous EOC has previously reported improved 5‐year OS compared to serous EOC.^35^ Data on the impact of histological subtype and MHT use following OC surgery is sparse. Histological subtype, serous versus non‐serous EOCs, was included as a variable in the regression analysis. Histological subtype was found to be significantly associated with survival rates in the univariate regression analysis. However, in the multivariable analysis it was no longer significant and was therefore not included. In a previous study by Power et al. exploring MHT use after non‐serous EOC, no negative effect on overall and disease‐free survival (DFS) regarding MHT use was found.^13^

Borderline ovarian tumors have an excellent 5‐year OS of 97% although there have been concerns regarding the effect of MHT use after serous BOTs.^2^ Due to few events during the follow‐up in our study, we were unable to analyze the histological subtype of BOTs separately. Only one study has previously tried to evaluate MHT use following treatment for BOT.^10^ In the prospective study by Mascharenas et al. they identified 150 women age 50–74 diagnosed with a BOT and found no indication that MHT use postdiagnosis was associated with deaths. So far, there is no evidence that MHT would be unsafe for this group of women. In our study including women with both premature and early menopause after treatment of BOTs we found that as many as 35.1% were non‐users, which indicates an undertreatment.

NEOCs are biologically a heterogeneous group of tumors. For many of the NEOCs, like BOTs, survival rates are excellent and the health benefits of MHT use among young women treated for these tumors need to be considered.^36^ Unfortunately, due to the small number of NEOCs in our study cohort we were only able to perform descriptive analysis of MHT use in this group. Interestingly, only one death occurred in the NEOC group during the 10‐year follow‐up. Due to the absence of studies on MHT in NEOC, valid comparisons could not be made with our results. Granulosa cell tumors as well as Sertoli‐Leydig cell tumors express estrogen and progesterone receptors. Since they are likely to be hormone‐dependent, MHT use is not generally recommended to women diagnosed with these histological subtypes. However, for other histological subtypes among NEOCs, such as germ‐cell tumors, MHT may be used following surgically induced menopause.

It is well known that OC survivors suffer from fatigue, sleep disorders, cognitive deficits, vasomotor symptoms and sexual dysfunction.^18^, ^37^ Ovarian cancer has the highest mortality rate of all gynecological malignancies. However, for young women with an early stage EOC, NEOC or BOT the prognosis is much better and they are often cured from the tumor. Nevertheless, many young women with OC will not receive MHT. Moreover, women with more advanced disease may, with new treatments like PARP inhibitors, become long‐term cancer survivors. Offering MHT use for these women has the potential to increase their quality of life considerably by improving menopause related problems. There is evidence that MHT use after premature and early menopause has protective effects on cardiovascular disease and osteoporosis as well as a longer overall survival.^4^ To prospectively evaluate the long‐term beneficial effects of MHT use in women with OC following early menopause an even longer follow‐up would probably be needed.

The main strengths of our study are the nationwide population‐based design with validated data registers and excellent coverage, together with the long‐term follow‐up. In addition, detailed descriptive data on MHT dispensing was provided. The applied survival method takes into account that a woman can be a non‐user and a user at different timepoints, avoiding delayed MHT use from influencing the results in the non‐user group.

Limitations included the lack of variables suggested to be associated with survival: BMI, smoking, tumor hormone‐receptor and BRCA status, separation of the high‐grade and low‐grade serous carcinomas, alcohol consumption, or other comorbidities besides other malignancies. It would have been preferable to include these variables; however, they were not available during the study period. Nevertheless, as the cohort consists of young women with a median age of 45 years, other comorbidities are less likely to influence the results. Nor did we have data on socioeconomic factors. Data on recurrence were incomplete in the register, and therefore, DFS could not be assessed. MHT dispensing was used as a proxy for MHT use, but we do not know if the women used the MHT dispensed. The survival analysis only considered the first MHT dispensing and not the total amount of MHT used or the duration of use.

Our results provide new important knowledge on the safety of MHT use following surgical menopause and suggest that MHT is safe to use after OC treatment. Larger population‐based studies are needed to verify these findings, ideally separating MHT type and administration routes as well as histological subtypes in detail. Also, the effect of MHT use on the recurrence of disease needs to be clarified. RCT studies on the topic are unlikely to be feasible due to the small number of premenopausal patients and also ethical considerations of not providing MHT to a randomized group of OC survivors.

## 5 CONCLUSION

In this nationwide population‐based cohort of premenopausal women surgically treated for OC, including EOC, postoperative use of MHT was not associated with an impaired 10‐year OS compared to women who never used MHT. These findings provide important reassurance regarding the oncological safety of MHT and suggest that MHT may be a viable option for managing menopausal symptoms and improving quality of life in young OC survivors. Given the potential long‐term health benefits and the absence of adverse effects on survival, MHT use should be considered in OC survivors with early menopause after treatment.

## AUTHOR CONTRIBUTIONS

ÅE: Conceptualization, data curation, and methodology. Formal analysis and interpretation of data. Writing original draft, and writing review and editing manuscript. PDK: Conceptualization, data curation, and methodology. Formal analysis and interpretation of data. Writing original draft, and writing review and editing manuscript. KRW: Conceptualization, interpretation of data, review, and editing manuscript. EH: Data curation and methodology. Formal analysis and methodology and interpretation of data. Writing original draft, and writing review and editing manuscript. AFR: Conceptualization, data curation, and methodology. Formal analysis and interpretation of data. Writing original draft, and writing review and editing manuscript. Funding acquisition.

## FUNDING INFORMATION

This project was funded by the Stockholm County Council and Karolinska Institutet (ALF Grant number 502434).

## CONFLICT OF INTEREST STATEMENT

The authors declare no conflicts of interest.

## ETHICS STATEMENT

The study was approved by the Regional Ethical Review Board in Stockholm Dnr 2016/1161‐31 (July 25, 2016), and amendments Dnr 2016/2189‐32 (December 7, 2016), Dnr 2017/1199‐32 (February 15, 2017), Dnr 2021‐01319 (April 22, 2021), Dnr 2025‐03758‐02 (May 31, 2025).

## Supporting information


**Table S1.** Patient, tumor, and treatment characteristics of epithelial ovarian cancer with no residual disease at surgery, stratified by MHT use within 5 years.

**Table S2.** Patient, tumor, and treatment characteristics of borderline ovarian tumors and non‐epithelial ovarian cancer stratified by diagnosis and MHT use within 5 years.

## Data Availability

The data that support the findings of this study are available from the corresponding author upon reasonable request.
