# Supplementary material for: Menopausal hormone therapy after ovarian cancer: A 10‐year survival analysis in premenopausal women
Source: Acta Obstet Gynecol Scand. 2026 May 20;105(8):1468–79. doi: 10.1111/aogs.70242 (PMC13356480; doi:10.1111/aogs.70242)
Supplement: Supplementary file 1 — Table S1. Patient, tumor, and treatment characteristics of epithelial ovarian cancer with no residual disease at surgery, stratified by MHT use within 5 years. [file AOGS-105-1468-s001.docx]

Table S1. Patient, tumor, and treatment characteristics of epithelial ovarian cancer with no residual disease at surgery, stratified by MHT use within five years.

|  |  | **MHT-user within 5 years** | |  |
| --- | --- | --- | --- | --- |
|  | **Total** | **No** | **Yes** | **p-value*** |
|  | **N=367** | **N=242 (65.9%)** | **N=125 (34.1%)** |  |
| Age at surgery (years), median (IQR) | 45.2 (41.0-48.2) | 47.0 (43.0-49.0) | 42.0 (38.0-46.1) | <0.001 |
| Age at surgery (years), age group |  |  |  | <0.001 |
| 18-29 | 18 | 12 (66.7) | 6 (33.3) |  |
| 30-34 | 14 | 4 (28.6) | 10 (71.4) |  |
| 35-39 | 45 | 18 (40.0) | 27 (60.0) |  |
| 40-44 | 84 | 47 (56.0) | 37 (44.0) |  |
| 45-50 | 206 | 161 (78.2) | 45 (21.8) |  |
| Year of surgery |  |  |  | 0.76 |
| 2008 | 36 | 23 | 13 |  |
| 2009 | 39 | 25 | 14 |  |
| 2010 | 58 | 41 | 17 |  |
| 2011 | 33 | 22 | 11 |  |
| 2012 | 26 | 20 | 6 |  |
| 2013 | 62 | 41 | 21 |  |
| 2014 | 61 | 35 | 26 |  |
| 2015 | 52 | 35 | 17 |  |
| FIGO stage |  |  |  | 0.088 |
| I | 172 | 104 (60.5) | 68 (39.5) |  |
| II | 44 | 35 (79.5) | 9 (20.5) |  |
| III | 122 | 82 (67.2) | 40 (32.8) |  |
| IV | 29 | 21 (72.4) | 8 (27.6) |  |
| Subtype histology |  |  |  | 0.25 |
| EOC Serous | 177 | 120 (67.8) | 57 (32.2) |  |
| EOC Mucinous | 57 | 35 (61.4) | 22 (38.6) |  |
| EOC Endometroid | 75 | 53 (70.7) | 22 (29.3) |  |
| EOC Clear Cell | 45 | 24 (53.3) | 21 (46.7) |  |
| EOC Other | 13 | 10 (76.9) | 3 (23.1) |  |
| Surgery type |  |  |  | 0.055 |
| Primary debulking surgery | 297 | 203 | 94 |  |
| Interval debulking surgery | 4 | 1 | 3 |  |
| Re-staging | 66 | 38 | 28 |  |
| Hysterectomy |  |  |  | 0.089 |
| No | 26 | 13 | 13 |  |
| Yes | 339 | 227 | 112 |  |
| Undefined | 2 | 2 | 0 |  |
| Adjuvant chemotherapy |  |  |  | 0.96 |
| Yes | 296 | 195 | 101 |  |
| No | 71 | 47 | 24 |  |
| Time to first MHT use (months), median (IQR) | 2.7 (0.3-8.4) |  | 2.7 (0.3-8.4) |  |
| Follow-up censored, median (IQR), years | 11.2 (9.5-13.6) | 11.3 (9.6-13.5) | 11.1 (9.3-13.7) | 1.00 |
| Follow-up death, median (IQR), years | 3.7 (2.3-6.1) | 3.3 (2.0-6.5) | 4.9 (2.8-6.0) | 0.24 |
| Number of death/censored within 5 years |  |  |  | 0.10 |
| Death | 86 | 63 | 23 |  |
| Censored | 281 | 179 | 102 |  |

MHT; menopausal hormone therapy

EOC; epithelial ovarian cancer

Data are presented as median (IQR) for continuous measures, and No. (row% %) for categorical measures.
*Comparisons between MHT groups were performed using Fisher's exact test for categorical variables and the Wilcoxon rank-sum test for continuous variables. Undefined, missing, or not applicable categories were excluded from statistical comparisons.
