# Supplementary material for: Menopausal hormone therapy after ovarian cancer: A 10‐year survival analysis in premenopausal women
Source: Acta Obstet Gynecol Scand. 2026 May 20;105(8):1468–79. doi: 10.1111/aogs.70242 (PMC13356480; doi:10.1111/aogs.70242)
Supplement: Supplementary file 2 — Table S2. Patient, tumor, and treatment characteristics of borderline ovarian tumors and non‐epithelial ovarian cancer stratified by diagnosis and MHT use within 5 years. [file AOGS-105-1468-s002.docx]

Table S2. Patient, tumor, and treatment characteristics of borderline ovarian tumors and non-epithelial ovarian cancer stratified by diagnosis and MHT use within 5 years

|  |  | **MHT-user within 5 years BOT** | |  |  | **MHT-user within 5 years NEOC** | |  |
| --- | --- | --- | --- | --- | --- | --- | --- | --- |
|  | **Total BOT** | **No** | **Yes** | **p-value*** | **Total NEOC** | **No** | **Yes** | **p-value*** |
|  | ***n*=239** | ***n*=84 (35.1%)** | ***n*=155 (64.9%)** |  | ***n*=68** | ***n*=31 (45.6%)** | ***n*=37 (54.4%)** |  |
| Age at surgery (years),  median (IQR) | 44.2 (39.0-48.0) | 47.0 (43.1-49.0) | 43.0 (37.1-46.0) | <0.001 | 39.6 (30.0-45.0) | 44.0 (33.0-48.0) | 37.0 (26.3-42.3) | 0.017 |
| Age at surgery (years), age group |  |  |  | <0.001 |  |  |  | 0.13 |
| 18-29 | 15 | 2 (13.3) | 13 (86.7) |  | 17 | 6 (35) | 11 (65) |  |
| 30-34 | 11 | 2 (18.2) | 9 (81.8) |  | 11 | 4 (36) | 7 (64) |  |
| 35-39 | 40 | 3 ( 7.5) | 37 (92.5) |  | 6 | 2 (33) | 4 (67) |  |
| 40-44 | 55 | 16 (29.1) | 39 (70.9) |  | 16 | 6 (38) | 10 (62) |  |
| 45-50 | 118 | 61 (51.7) | 57 (48.3) |  | 18 | 13 (72) | 5 (28) |  |
| Year of surgery |  |  |  | 0.42 |  |  |  | 0.42 |
| 2008 | 24 | 8 | 16 |  | 5 | 1 | 4 |  |
| 2009 | 31 | 15 | 16 |  | 8 | 3 | 5 |  |
| 2010 | 36 | 11 | 25 |  | 13 | 6 | 7 |  |
| 2011 | 32 | 8 | 24 |  | 9 | 7 | 2 |  |
| 2012 | 29 | 9 | 20 |  | 5 | 1 | 4 |  |
| 2013 | 23 | 7 | 16 |  | 10 | 4 | 6 |  |
| 2014 | 33 | 16 | 17 |  | 11 | 6 | 5 |  |
| 2015 | 31 | 10 | 21 |  | 7 | 3 | 4 |  |
| FIGO stage |  |  |  | 0.049 |  |  |  | 0.088 |
| I | 202 | 78 (38.6) | 124 (61.4) |  | 61 | 31 (51) | 30 (49) |  |
| II | 10 | 2 (20.0) | 8 (80.0) |  | 3 | 0 ( 0) | 3 (100) |  |
| III | 26 | 4 (15.4) | 22 (84.6) |  | 3 | 0 ( 0) | 3 (100) |  |
| IV | 1 | 0 ( 0.0) | 1 (100) |  | 1 | 0 ( 0) | 1 (100) |  |
| Subtype histology |  |  |  | 0.61 |  |  |  |  |
| BOT Serous | 61 | 18 (29.5) | 43 (70.5) |  |  |  |  |  |
| BOT Mucinous | 67 | 25 (37.3) | 42 (62.7) |  |  |  |  |  |
| BOT Other | 111 | 41 (36.9) | 70 (63.1) |  |  |  |  |  |
|  |  |  |  |  |  |  |  | 0.002 |
| NEOC Germ Cell |  |  |  |  | 22 | 4 (18) | 18 (82) |  |
| NEOC Sex Cord-Stromal Cell |  |  |  |  | 46 | 27 (59) | 19 (41) |  |
| Type of surgery |  |  |  | 0.26 |  |  |  | 0.31 |
| Primary debulking surgery (PDS) | 181 | 68 | 113 |  | 39 | 20 | 19 |  |
| Interval debulking surgery (IDS) | 1 | 0 | 1 |  |  |  |  |  |
| Re-staging | 49 | 13 | 36 |  | 26 | 10 | 16 |  |
| Undefined/missing | 8 | 3 | 5 |  | 3 | 1 | 2 |  |
| Complete cytoreduction to no residual disease |  |  |  | 0.61 |  |  |  | 0.67 |
| Yes | 216 | 73 (33.8) | 143 (66.2) |  | 59 | 27 (46) | 32 (54) |  |
| No | 4 | 2 (50.0) | 2 (50.0) |  | 3 | 1 (33) | 2 (67) |  |
| Undefined/missing/not applicable | 19 | 9 (47.4) | 10 (52.6) |  | 6 | 3 (50) | 3 (50) |  |
| Hysterectomy |  |  |  | 1.00 |  |  |  | 0.57 |
| No | 23 | 8 | 15 |  | 26 | 13 | 13 |  |
| Yes | 216 | 76 | 140 |  | 42 | 18 | 24 |  |
| Adjuvant chemotherapy |  |  |  | 0.005 |  |  |  | 0.049 |
| Yes | 32 | 4 | 28 |  | 33 | 11 | 22 |  |
| No | 207 | 80 | 127 |  | 35 | 20 | 15 |  |
| Time to first MHT use (months), median (IQR) | 1.1 (0.1-3.5) |  | 1.1 (0.1-3.5) |  | 5.2 (0.8-12.6) |  | 5.2 (0.8-12.6) |  |
| Follow-up censored, median (IQR), years | 11.8 (9.7-13.6) | 11.8 (9.6-13.9) | 11.9 (9.8-13.6) | 0.91 | 12.1 (9.9-13.4) | 12.2 (9.9-12.9) | 11.8 (9.9-13.7) | 0.32 |
| Follow-up death, median (IQR), years | 6.6 (4.5-8.3) | 6.8 (3.6-8.8) | 6.3 (5.1-7.9) | 0.94 | 8.0 (0.6-15.4) | 8.0 (0.6-15.4) |  |  |
| Number of deaths/censored within 5 years |  |  |  | 0.28 |  |  |  | 0.27 |
| Death | 3 | 2 (66.7) | 1 (33.3) |  | 1 | 1 (100) | 0 ( 0) |  |
| Censored | 236 | 82 (34.7) | 154 (65.3) |  | 67 | 30 (45) | 37 (55) |  |

MHT; menopausal hormone therapy BOT; borderline ovarian tumor

NEOC; non-epithelial ovarian cancer

Data are presented as median (IQR) for continuous measures, and No. (row% %) for categorical measures.
*Comparisons between MHT groups were performed using Fisher's exact test for categorical variables and the Wilcoxon rank-sum test for continuous variables. Undefined, missing, or not applicable categories were excluded from statistical comparisons.
